# Antiglycation approach in selected autoimmune diseases: Where do we stand?

**DOI:** 10.1016/j.redox.2026.104410

**Published:** 2026-09-22

**Authors:** Szymon Drygała, Roman Cemaga, Mateusz Maciejczyk

**Affiliations:** a Department of Hygiene, Epidemiology and Environmental Health, Medical University of Bialystok, 2c Mickiewicza Street, Bialystok, 15-222, Poland; b Students Scientific Club “Biochemistry of Lifestyle Diseases” at the Department of Hygiene, Epidemiology and Environmental Health, Medical University of Bialystok, 2c Mickiewicza Street, Bialystok, 15-222, Poland

**Keywords:** AGE, RAGE, Autoimmunity, Rheumatoid arthritis, Systemic lupus erythematosus, Type 1 diabetes, Antiglycation

## Abstract

Advanced glycation end products (AGEs) and their receptor for advanced glycation end products (RAGE) have emerged as potential contributors to autoimmune pathology by linking metabolic stress, oxidative injury, and chronic inflammation. This review summarizes current mechanistic, preclinical, and clinical evidence on the role of the AGE–RAGE axis in selected autoimmune diseases, with a focus on systemic lupus erythematosus, rheumatoid arthritis, type 1 diabetes, psoriasis, and multiple sclerosis. Across these conditions, increased AGE accumulation, enhanced availability of RAGE ligands, and altered soluble RAGE buffering appear to be recurring features associated with disease activity, organ damage, vascular complications, and immune dysregulation. Mechanistically, AGE–RAGE signaling amplifies nuclear factor kappa B (NF-κB), mitogen-activated protein kinase (MAPK), Janus kinase/signal transducer and activator of transcription (JAK/STAT), and reactive oxygen species (ROS)-dependent pathways, promotes pro-inflammatory immune phenotypes, and may contribute to neoepitope generation and epitope spreading. We also discuss anti-glycation strategies, including carbonyl scavengers, AGE formation inhibitors, redox-modulating agents, crosslink breakers, and RAGE-targeting interventions. Although several compounds show promising anti-inflammatory and antiglycation effects in experimental models, autoimmune-specific translational evidence remains limited, and most studies do not include validated AGE/RAGE biomarkers. Overall, the AGE–RAGE axis represents a plausible therapeutic target in autoimmunity, but establishing its therapeutic potential will require standardized biomarker panels and well-designed clinical trials.

## 1 Introduction

Autoimmune diseases represent a heterogeneous group of chronic disorders characterized by an aberrant immune response directed against self-antigens, leading to tissue inflammation and organ damage. More than 80 distinct autoimmune conditions have been identified, affecting approximately 5–8% of the global population, with a higher prevalence in women [1,2]. Although these diseases vary in clinical presentation and target tissues, they share common immunopathological features, including the breakdown of immune tolerance, autoreactive lymphocyte activation, production of autoantibodies, and sustained inflammatory signaling [2]. While genetic predisposition and environmental factors are well-established contributors, increasing attention has been given to the role of metabolic stress and post-translational protein modifications in shaping immune responses [[3], [4], [5], [6], [7], [8]]. Altered energy metabolism in autoreactive lymphocytes, enhanced reactive oxygen species (ROS) production, and accumulation of reactive dicarbonyl compounds (e.g., methylglyoxal (MGO)) contribute to a proinflammatory milieu [9]. These metabolic perturbations link traditional immune dysregulation with biochemical processes such as protein glycation, positioning advanced glycation end products (AGEs) and their receptor (RAGE) as potential amplifiers of autoimmune pathology [[10], [11], [12], [13]]. AGEs are a diverse class of biomolecular modifications arising from non-enzymatic reactions between reducing sugars or reactive carbonyl species and nucleophilic amino groups in proteins, lipids, and nucleic acids. This non-enzymatic chemistry is commonly described as the Maillard reaction, which operates spontaneously under physiological conditions but is markedly accelerated by hyperglycemia and oxidative stress [[14], [15], [16]]. Glycation proceeds through early-stage Schiff base formation and Amadori rearrangement, followed by late-stage oxidation and carbonyl-driven reactions that generate largely irreversible AGEs, including non-crosslinking adducts (carboxymethyllysine (CML) and carboxyethyllysine (CEL)), and crosslinking structures (pentosidine) [17,18]. Early glycation adducts can be partially reversible and serve as substrates for further glycoxidation, whereas late AGEs accumulate and can act as proinflammatory damage-associated molecular pattern (DAMP)-like ligands that engage RAGE and other scavenger receptors [19,20]. AGEs, such as CML, CEL, and pentosidine can alter protein conformation, induce oxidative stress, and trigger inflammatory signaling, thereby linking glycative chemistry to a broad spectrum of disease processes [[21], [22], [23]]. AGEs act via RAGE, which belongs to the immunoglobulin superfamily and acts as a transmembrane pattern recognition receptor that recognizes a broad range of endogenous and exogenous ligands [18,24,25]. The wide spectrum of receptor ligands makes it a central hub in processes such as inflammation, metabolic disorders, aging, cancer, and tissue remodeling [18,19]. The soluble form of RAGE (sRAGE), generated by alternative splicing or proteolytic shedding, can act as a decoy receptor that sequesters ligands, and thereby blunts RAGE-mediated signaling [[26], [27], [28]]. Beyond the canonical lysine-derived adducts CML and CEL and fluorescent crosslinks such as pentosidine, carbonyl stress also modifies cysteine residues. Glyoxal can react with protein thiols to generate the stable advanced glycation product S-(carboxymethyl)cysteine (CMC), whereas methylglyoxal-modified human serum albumin has been shown to contain S-(carboxyethyl)cysteine (CEC) together with lysine- and arginine-derived adducts [29,30]. This chemistry is particularly relevant to redox biology because albumin constitutes a major extracellular antioxidant system through its reactive thiol pool. Methylglyoxal-dependent thiol modification can decrease albumin antioxidant capacity [31]. CMC and CEC therefore provide a mechanistically relevant link between dicarbonyl stress, depletion of protein thiols, and impaired extracellular redox buffering, although they are currently measured far less frequently than CML, CEL, or pentosidine in autoimmune cohorts [29].

Disruption of AGE–RAGE signaling alleviates inflammatory signaling and tissue remodeling, supporting the translational viability of anti-glycation interventions to modulate autoimmune diseases [7,32]. AGEs may directly contribute to autoimmunity by generating glycated neoepitopes that broaden B-and T-cell repertoires and facilitate epitope spreading [7,32], stiffening extracellular matrix through AGE crosslinking, which can potentiate an inflammation [33], and amplifying innate immune activation via RAGE-dependent NF-κB/MAPK signaling and ROS production [20,34]. In tissues exposed to chronic inflammation, AGE accumulation can integrate metabolic stress to sustain a feed-forward inflammatory loop [35,36]. Taken together, the coherence of AGE–RAGE signaling with autoimmune pathophysiology there is a clear rationale to investigate anti-glycation and RAGE-targeted strategies as disease modifying entry points in autoimmune diseases. This review aims to synthesize current mechanistic, preclinical, and clinical evidence and to explore the therapeutic potential of targeting the AGE–RAGE axis in systemic autoimmune diseases, including systemic lupus erythematosus (SLE), rheumatoid arthritis (RA), type 1 diabetes (T1D), psoriasis, and multiple sclerosis (MS).

## 2 RAGE biology: structure, expression, ligands, and soluble forms

RAGE is a type I single-pass transmembrane pattern-recognition receptor belonging to the immunoglobulin superfamily. The extracellular ectodomain comprises one N-terminal variable-type immunoglobulin-like domain (V domain) followed by two constant-type immunoglobulin-like domains (C1 and C2) [37]. Structural and biophysical studies indicate that the V and C1 domains form a relatively compact, functionally integrated VC1 unit, whereas the C2 domain is connected to C1 through a more flexible linker and displays substantially greater conformational freedom [38]. This architecture is relevant to ligand promiscuity. The positively charged and hydrophobic surfaces of the V and C1 domains constitute a major ligand-recognition platform for several structurally unrelated RAGE ligands, although individual ligands can engage distinct combinations of the V, C1, and C2 domains [39]. Thus, RAGE should not be conceptualized as possessing a single conventional ligand-binding pocket. Rather, it recognizes molecular patterns generated by structurally heterogeneous, frequently oligomeric or negatively charged ligands, allowing several classes of damage-associated molecular patterns to converge on the same receptor. [37,40]. RAGE can exist in preassembled or ligand-stabilized oligomeric states, and receptor clustering is increasingly considered an important determinant of productive signal transduction. Cell-surface heparan sulfate can stabilize higher-order RAGE complexes and has been shown to be required for efficient signaling by several RAGE ligands, providing an additional level of regulation beyond simple ligand–receptor affinity [41]. The cytoplasmic domain subsequently recruits intracellular adaptor proteins. Of particular importance is diaphanous-related formin-1 (DIAPH1), which directly interacts with RAGE cytoplasmic tail and is required for several ligand-induced cellular responses, including activation of Rac1 and Cdc42 and regulation of cellular migration. Mutational disruption of the DIAPH1-interacting region of the cytoplasmic tail markedly attenuates ligand-dependent RAGE signaling, supporting the concept that RAGE–DIAPH1 interface constitutes a proximal signaling node rather than the cytoplasmic tail functioning merely as a passive membrane anchor [41]. Depending on cell type and ligand context, downstream signaling can involve NF-κB, extracellular signal-regulated kinases 1/2 (ERK1/2), p38 and c-Jun N-terminal kinase (JNK) MAPKs, phosphoinositide 3-kinase/protein kinase B (PI3K/AKT), JAK/STAT pathways, small GTPases, and nicotinamide adenine dinucleotide phosphate (NADPH) oxidase-dependent ROS generation. Importantly, activation of NF-κB may additionally enhance advanced glycosylation end-product specific receptor (AGER) transcription, creating a positive-feedback circuit in which ligand accumulation increases RAGE expression and thereby sensitizes cells to continued extracellular danger signaling [19,36]. Beyond AGEs, well-established endogenous RAGE ligands include high mobility group box 1 (HMGB1) and several members of the S100 family, including S100B, S100A8/A9 and S100A12 [37,40]. Ligand recognition is domain-specific to some extent: several ligands predominantly interact with the V or VC1 region, whereas selected S100 proteins can additionally use C1- and/or C2-containing binding surfaces. RAGE also interacts with β-amyloid and other β-sheet-rich assemblies and contributes to amyloid-β transport and accumulation at the blood–brain barrier [39,42]. Moreover, experimental studies have demonstrated direct interaction with bacterial lipopolysaccharide (LPS), contributing to LPS-associated inflammatory responses and septic shock [43]. Because LPS is simultaneously a canonical Toll-like receptor 4 (TLR4) ligand, this observation also illustrates the broader principle that RAGE frequently operates within interconnected pattern-recognition networks rather than as an isolated signaling system. Crosstalk with Toll-like receptors (TLRs) and convergence at NF-κB/MAPK and ROS-generating pathways may therefore amplify inflammatory responses initiated by either metabolic DAMPs or microbial products.

Circulating soluble RAGE (sRAGE) denotes a heterogeneous pool of molecules that retain the extracellular ligand-binding domains but lack the transmembrane and signaling-competent cytoplasmic regions [44]. At least two principal mechanisms contribute to this pool. Endogenous secretory RAGE (esRAGE) is generated by alternative splicing of AGER pre-mRNA and is secreted without having been expressed as a full-length membrane receptor [45]. In contrast, cleaved RAGE (cRAGE) is generated by proteolytic ectodomain shedding of membrane-bound RAGE. A disintegrin and metalloproteinase 10 (ADAM10) represents a major RAGE sheddase, although matrix metalloproteinases (MMPs) and other proteolytic mechanisms may also contribute depending on cellular context [46]. Both soluble forms retain the extracellular ligand-binding region while lacking the transmembrane and cytoplasmic domains required for conventional RAGE signal transduction. Consequently, they can compete with membrane-bound RAGE for extracellular ligands and thereby attenuate ligand-dependent receptor activation [47,48]. Nevertheless, circulating sRAGE concentrations do not provide a direct quantitative readout of membrane RAGE activity. Inflammatory and oxidative conditions may simultaneously enhance tissue RAGE expression and stimulate metalloproteinase-dependent ectodomain shedding, thereby increasing the circulating cRAGE fraction despite ongoing receptor activation [49]. Moreover, serum sRAGE is influenced by factors unrelated to receptor signaling intensity, including renal function, metabolic status and pharmacological exposure. Reduced renal clearance, may increase circulating sRAGE concentrations independently of enhanced decoy activity [49,50]. Thus, higher sRAGE may represent compensatory ligand sequestration in one setting but increased receptor turnover, inflammatory protease activity or impaired clearance in another. Conversely, low sRAGE may be consistent with diminished extracellular ligand buffering, but in isolation does not demonstrate enhanced membrane-RAGE signaling. Separate quantification of esRAGE and cRAGE may therefore provide greater mechanistic resolution than measurement of total sRAGE alone.

## 3 Pathogenic mechanisms of the AGE–RAGE axis in autoimmunity

The AGE–RAGE axis acts as a potent amplifier of inflammatory signaling in autoimmunity, integrating metabolic stress with innate immune sensing to sustain chronic inflammation that can drive upregulation of autoimmune pathology [20,51,52]. AGE engagement with RAGE initiates multiple parallel intracellular signaling cascades: JAK–STAT, PI3K–AKT, MAPK cascade (including ERK1/2, JNK, and p38), and the NADPH oxidase–ROS pathways. Additionally, NF-κB signaling is a central downstream mediator of AGE–RAGE signaling, frequently activated via cross-talk with these cascades [53]. Meaningful additional interactions include HMGB1–RAGE signaling, cross-talk with TLR4, Notch signaling, protein kinase C (PKC) pathways, and activation of the nucleotide-binding oligomerization domain-like receptor family pyrin domain-containing protein 3 (NLRP3) inflammasome [10,15,[54], [55], [56], [57]]. The inflammatory amplification is reinforced by ROS and mitochondrial dysfunction, creating a feed-forward loop that perpetuates tissue damage and sustains autoimmune response [2,58]. Experimental models showed that AGE–RAGE engagement drives naive cluster of differentiation 4-positive (CD4^+^) T cells to differentiate toward pro-inflammatory T helper 1/T helper 17 (Th1/Th17) phenotypes and impairs the suppressive function of regulatory T cells (Tregs), and activates macrophage activation, which are both drivers of autoimmunity [[59], [60], [61]] (Fig. 1). Activation of discussed pathways leads to increased transcription of cytokines like interleukin 1 beta (IL-1β), interleukin 6 (IL-6), and tumor necrosis factor alpha (TNF-α), and upregulation of adhesion molecules (e.g., intercellular adhesion molecule 1 (ICAM-1), vascular cell adhesion molecule 1 (VCAM-1)), which together create a pro-inflammatory environment that promotes autoimmunity [34,53]. The cumulative effect is a robust, self-sustaining pro-inflammatory axis in which RAGE serves as a central hub that perpetuates autoimmune responses (Table 1).

**Fig. 1:**
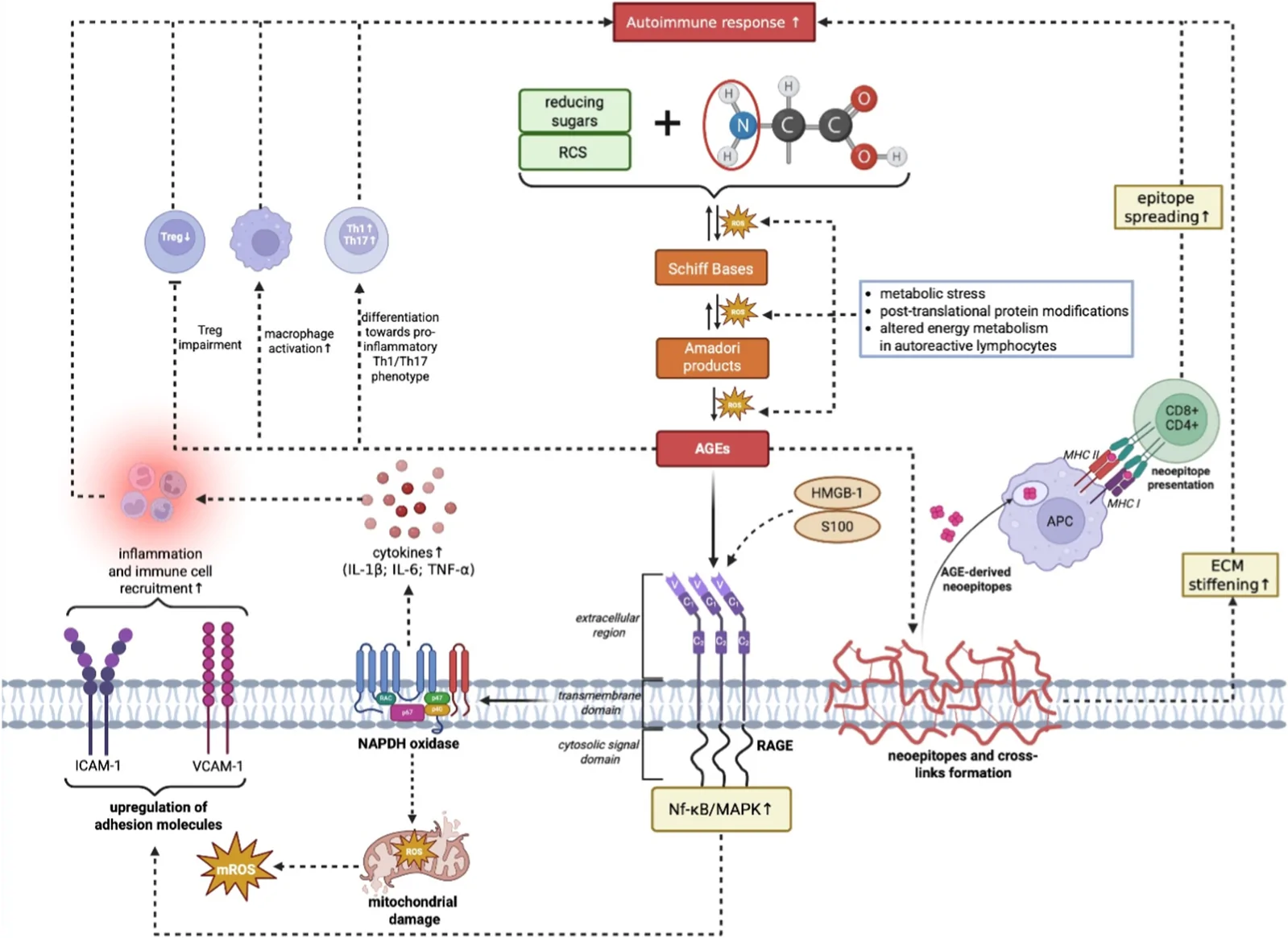
**Proposed pathogenic mechanisms linking the AGE–RAGE axis to autoimmune responses.** This schematic summarizes the formation of advanced glycation end products (AGEs) from reducing sugars and reactive carbonyl species (RCS), and the downstream consequences of AGE accumulation in autoimmunity. AGE–RAGE signaling promotes NF-κB/MAPK activation, oxidative stress, cytokine production, endothelial activation, and immune-cell recruitment, while also contributing to macrophage activation, Th1/Th17 skewing, impaired regulatory T-cell function, neoepitope formation, epitope spreading, and extracellular matrix stiffening. Together, these processes may amplify chronic inflammation and sustain autoimmune responses. Abbreviations: AGE, advanced glycation end product; APC, antigen-presenting cell; C_1_, C_1_ domain of RAGE; C_2_, C_2_ domain of RAGE; ECM, extracellular matrix; HMGB1, high mobility group box 1; ICAM-1, intercellular adhesion molecule 1; MAPK, mitogen-activated protein kinase; mROS, mitochondrial reactive oxygen species; NF-κB, nuclear factor kappa B; RAGE, receptor for advanced glycation end products; RCS, reactive carbonyl species; ROS, reactive oxygen species; VCAM-1, vascular cell adhesion molecule 1; V, V domain of RAGE.

**Table 1:** Key signaling nodes and immune outcomes downstream of AGE–RAGE in autoimmunity.

AGE–RAGE-linked mechanism	Major inflammatory pathways	Functional immune outcomes	Representative readouts (examples)	Key References
Inflammatory signal amplification	JAK–STAT; PI3K–AKT; MAPK (ERK1/2, JNK, p38); NADPH oxidase → ROS; NF-κB as central integrator	Sustained innate activation; persistence of sterile inflammation	↑ IL-1β, IL-6, TNF-α; ↑ ICAM-1/VCAM-1	[20,34,[51], [52], [53]]
Crosstalk with DAMP signaling	HMGB1–RAGE; S100–RAGE; interaction with TLR signaling	Reinforced inflammatory tone; enhanced leukocyte recruitment	↑ chemotaxis signals; ↑ macrophage/neutrophil influx	[35,37,62]
Oxidative stress feed-forward loop	NADPH oxidase activation; mitochondrial ROS (mROS); impaired antioxidant capacity	Self-perpetuating inflammatory cycle; tissue injury	↑ ROS/mROS; mitochondrial dysfunction markers	[51,60]
Adaptive skewing toward pathogenic T-cell phenotypes	RAGE-driven inflammatory milieu supports T-cell polarization	Th1/Th17 bias; reduced Treg suppressive capacity	↑ Th1/Th17 cytokine signatures; ↓ Treg function	[51,60]
Macrophage activation as an effector amplifier	RAGE activation in macrophages (ligand dependent)	Enhanced inflammatory effector activity	↑ cytokines; ↑ oxidative enzymes	[59,63]

AGE – Advanced Glycation End-Products; AKT – Protein Kinase B; DAMP – Damage Associated Molecular Pattern; ERK 1/2 – Extracellular Signal Regulated Kinase 1/2; HMGB1 – High Mobility Group Box 1; ICAM-1 – Intercellular Adhesion Molecule 1; IL-1β – Interleukin 1-beta; IL-6 – Interleukin 6; JAK – Janus Kinase; JNK – c-Jun N-terminal Kinase; MAPK – Mitogen Activated Protein Kinase; mROS – mitochondrial reactive oxygen species; NADPH – Nicotinamide Adenine Dinucleotide Phosphate; NF-κB – Nuclear Factor kappa B; p38 – p38 Mitogen Activated Protein Kinase; PI3K – Phosphoinositide 3-Kinase; RAGE – Receptor for Advanced Glycation End Products; ROS – Reactive Oxygen Species; S100 – S100 Proteins; STAT – Signal Transducer and Activator of Transcription; Th1/Th17 – Lymphocyte T helper type 1/17; TLR – Toll Like Receptor; TNF-α – Tumor Necrosis Factor alpha; Treg – Regulatory T cells; VCAM-1 – Vascular Cell Adhesion Molecule 1.

As discussed earlier, RAGE functions as a multi-ligand pattern recognition receptor, which interacts not only with AGEs, but also with damage-associated molecular patterns (DAMPs) such as high mobility group box 1 (HMGB1) and S100/calgranulin proteins [37,64,65]. DAMPs released by stressed or damaged tissues bind to RAGE, thereby escalating inflammatory signals initiated by AGEs. This interaction establishes a feedback loop in which RAGE signaling remains persistently activated by both AGE and DAMP ligands. Sustained receptor engagement promotes continuous activation of pro-inflammatory pathways and facilitates the recruitment of immune cells, including macrophages and neutrophils. Consequently, a robust sterile inflammatory milieu is maintained, which can persist in the context of autoimmune conditions [35,53,62]. Furthermore, sustained activation of RAGE by concurrent AGE and DAMP ligands is associated with cellular dysfunction and apoptosis, underscoring RAGE's broader impact in autoimmune pathologies beyond acute inflammation [53].

Oxidative stress and mitochondrial dysfunction are both cause and consequence of AGE–RAGE signaling. Engagement of RAGE by AGEs or other ligands leads to increased ROS generation via NADPH oxidase and mitochondrial sources, fostering a proinflammatory milieu [36,63,66,67]. In models of metabolic disease and autoimmunity, RAGE activation correlates with elevated ROS production, inflammatory cytokines, and vascular/endothelial injury, supporting a feed-forward loop in which oxidative stress amplifies RAGE signaling and vice versa [66,68]. Mitochondrial dysfunction is both a driver and a substrate of this loop. Mitochondria are particularly susceptible to ROS, and AGE–RAGE signaling can impair mitochondrial function, reduce antioxidant capacity, and exacerbate mitochondrial ROS (mROS) production, which further fueling the cycle and contributing to tissue damage [67,69,70]. In rheumatoid arthritis and systemic lupus erythematosus, mitochondrial impairment and defective mitophagy are recognized as contributors to disease activity and tissue damage [68,[71], [72], [73]] (Table 1).

Recently, the neoantigen hypothesis has been introduced as a conceptual framework for understanding the initiation and progression of autoimmune disorders. This hypothesis proposes that autoimmunity originates not from inherent defects in immune regulation, but rather from the generation of neoantigens through disease-specific molecular processes [74]. Neoantigens are disease-specific modified proteins, whereas neoepitopes are the major histocompatibility complex (MHC)-presented peptide fragments derived from those neoantigens. Neoepitopes arise through several mechanisms: 1) genetic alterations such as somatic mutations and gene fusion, 2) altered RNA processing and splicing yield splice-neoantigens and variations in epitopes, 3) non-canonical translation and fusion-derived neoantigens, and 4) post-translational modifications including citrullination, homocitrullination, and glycooxidative modifications. Glucose oxidation leads to the formation of glyoxal and methylglyoxal, which are very potent glycating factors [70,[75], [76], [77], [78]]. AGEs are known to exhibit antigenic properties that can stimulate autoimmune responses [32]. Moreover, glycation-induced modifications of human serum albumin and fibrinogen have been shown to generate neoepitopes that elicit strong autoimmune responses, thereby potentially contributing to the pathogenic mechanisms underlying conditions such as systemic lupus erythematosus (SLE) and rheumatoid arthritis (RA) [[79], [80], [81], [82], [83]]. Neoepitope driven autoimmunity is initiated when antigen-presenting cells (APCs) process and display neoepitopes to T cells, leading to autoreactive T cell responses against structurally altered self-determinants. APCs involved in this process include conventional dendritic cells and B cells, as well as tissue-specific and unconventional presenters that can present neoepitopes via MHC class I and II pathways to cluster of differentiation 8-positive (CD8^+^) and CD4^+^ T cells, respectively [[84], [85], [86]]. APC-driven neoepitope recognition can drive epitope spreading, whereby reactivity broadens from initial neoepitopes to additional self-antigens, intensifying autoimmunity and further decreasing immune tolerance [87,88](Fig. 1).

## 4 Disease-specific evidence in autoimmune conditions

### 4.1 Systemic lupus erythematosus (SLE)

SLE is a systemic autoimmune disease characterized by significant clinical heterogeneity encompassing mucocutaneous, musculoskeletal, renal, neuropsychiatric, hematologic, and cardiovascular domains [89]. The disease is driven by dysregulated innate and adaptive immune responses that culminate in chronic inflammation and tissue injury [90,91]. The autoimmune nature of SLE is highlighted by autoreactive B- and T-cell responses, impaired clearance of apoptotic debris, and dendritic-cell dysregulation that together sustain a self-perpetuating inflammatory environment [92]. TLR engagement and NF-κB/MAPK signaling create a self-amplifying cycle of inflammation that can perpetuate tissue injury across organ systems and disease stages [93]. In the context of glycation, this cycle is reinforced by inflammation-driven oxidative and carbonyl stress, which accelerates formation and tissue accumulation of AGEs. AGEs, together with other damage-associated ligands that accumulate during tissue injury (e.g., HMGB1 and S100/calgranulins), can activate RAGE, a multiligand pattern-recognition receptor that converges on pro-inflammatory signaling pathways including NF-κB and MAPKs [20,93]. This creates a loop in which TLR-driven inflammation promotes AGE generation and release of RAGE ligands, while AGE–RAGE signaling further amplifies NF-κB/MAPK activity, sustaining cytokine production, endothelial activation, and leukocyte recruitment [20,93,94]. In lupus-prone MRL/lpr mice, genetic deletion of Ager (RAGE) improved nephritis and reduced neutrophil infiltration and NET formation, supporting a role of RAGE signaling in renal inflammation [95]. The integration of the AGE-RAGE axis into SLE pathophysiology provides a mechanistic link between metabolic inflammation and autoimmunity [51,96,97].

Multiple studies link AGEs and their receptor axis to disease activity and redox imbalance in SLE, with several of them reporting detectable circulating AGEs in patients [98,99]. Some reports link AGE to clinical features and describe more moderate or inconsistent associations across cohorts, highlighting the clear heterogeneity in biomarker utility and the need to standardize tests and cohorts [96,[100], [101], [102]]. The mechanistic basis for AGE accumulation intersects with oxidative stress, as ROS drive glycation of proteins and lipids, yielding a spectrum of AGE structures that can persist in serum and tissues [33]. In inflammatory contexts such as active lupus nephritis (LN), these glycation processes are particularly relevant, given the close association between redox imbalance and organ involvement in SLE [103]. Although levels of MGO in SLE patients have not been conclusively defined, downstream AGEs formed from MGO are consistently observed in inflammatory settings, indicating their potential contribution to SLE pathology [104]. Collectively, the convergence of AGE accumulation with oxidative stress and RAGE signaling provides a plausible mechanistic framework for how metabolic stress may fuel autoimmunity and organ damage in SLE.

AGEs can serve as a potential biomarker for SLE. In a cross-sectional study including 55 patients with inactive SLE and 55 age and sex matched controls, AGE burden was quantified in serum using ultraviolet A (UV-A)–excited fluorescence emission matrices (autofluorescence–excitation/emission matrix spectroscopy, AF-EEMS), which captures the composite fluorescence signature of multiple fluorophore AGEs rather than individual adduct concentrations. SLE patients demonstrated significantly higher AF-EEMS values compared with controls (1.50 ± 0.5 a.u. vs. 1.28 ± 0.4 a.u.), indicating increased AGE-related signals even in inactive disease [99]. Similarly, in a cohort of 31 female SLE patients and 26 matched healthy women, AF-EEMS values were significantly elevated in the SLE group. However, specific AGE subtypes, including CML and CEL, did not differ between patients and controls, and were not influenced by disease activity [98]. Subsequent studies have provided further insights. Nowak et al. [96] reported significantly higher total serum AGE levels in SLE patients, although levels of CML, CEL, and pentosidine did not differ significantly from those of controls. Ene et al. [100] observed marked increases in both serum pentosidine and total AGE levels in SLE patients, irrespective of lupus nephritis status. Compared with controls, pentosidine concentrations were elevated by +249% in non-LN patients, +276% in LN patients, and +278% in type IV LN (severe, diffuse, proliferative glomerular lesions affecting more than 50% of the glomeruli), while total AGE levels were increased by +216%, +243%, and +267%, respectively. Notably, there were no significant differences between LN and non-LN subgroups. More recent investigations employing skin autofluorescence (SAF; a non-invasive measure of tissue AGEs) have demonstrated a robust association with disease activity. Carrión-Barberà et al. [101] reported significantly higher skin AGE levels in SLE patients compared with healthy controls. In the adjusted multiple linear regression model, AGEs values in SLE were 0.721 units higher than in healthy controls (HC) (95% confidence interval (CI) [0.566; 0.876], p < 0.001). Furthermore, skin AGE accumulation was associated with both the Systemic Lupus Erythematosus Disease Activity Index (SLEDAI) and the Systemic Damage Index (SDI), with progressively higher values observed in patients with moderate and severe disease activity and those with a high organ damage burden. Disease activity was also associated with elevated AGEs. Complementary findings were observed in serum measurements: pentosidine was linked to pulmonary involvement. CML was associated with anti-double-stranded DNA (anti-dsDNA) antibody titers, IL-6 levels, disease duration, and non-Caucasian ethnicity, and CEL was associated with anti-dsDNA, IL-6, and overall disease manifestations [102]. Finally, psychological comorbidity may also influence AGE levels. Żuchowski et al. [105] reported significantly higher skin AGE in SLE patients with comorbid depression compared to those without, suggesting that psychosocial factors may modulate AGE burden. Available evidence indicates that AGEs are consistently elevated in SLE compared with healthy controls. Associations with disease activity, organ damage, autoantibody status, inflammatory mediators, and specific clinical manifestations further support their potential utility as biomarkers. However, heterogeneity in findings regarding specific AGE subtypes (e.g., CML, CEL, pentosidine) and the influence of comorbid conditions (e.g., depression) highlight the need for standardized measurement approaches and longitudinal studies. AF-based approaches (AF-EEMS in serum; skin autofluorescence in vivo) are sensitive to fluorescent AGE structures (e.g., pentosidine-like crosslinks) but do not directly quantify non-fluorescent adducts (e.g., CML/CEL), which should be measured by targeted liquid chromatography–tandem mass spectrometry (LC–MS/MS) or immunoassays for mechanistic interpretation [8,98].

Soluble RAGE (sRAGE), the circulating isoform of RAGE, has been extensively investigated as a potential biomarker in SLE. As a decoy receptor, sRAGE neutralizes pro-inflammatory ligands, such as AGEs, S100 proteins, and HMGB1, thereby attenuating downstream NF-κB signaling [26]. Multiple cross-sectional studies demonstrate significantly reduced sRAGE levels in SLE compared to healthy controls, alongside elevated circulating or tissue AGE burden. In one of the earliest plasma studies, Ma et al. [94] reported that plasma sRAGE levels were significantly lower in 105 SLE patients and 43 healthy controls (p = 0.003). Notably, therapy influenced levels: short-term glucocorticoid treatment decreased sRAGE levels compared with untreated patients (p = 0.023), whereas long-term treatment increased sRAGE levels (p < 0.001), restoring concentrations toward those of healthy controls (p = 0.305). A cross-sectional study of 31 women with SLE and 26 female controls confirmed reduced serum sRAGE in patients [96]. Very similar findings were reported by Chen et al., [106] who found that plasma sRAGE levels were significantly lower in active SLE (n = 25) than in healthy controls (p < 0.05) and in inactive SLE (p < 0.05). Moreover, sRAGE levels correlated negatively with disease activity (SLEDAI; p < 0.005). Larger cohorts expanded these findings: in 119 SLE patients, Carrión-Barberà et al. [101] showed that serum sRAGE levels were associated with sex (lower in males, p < 0.05), photosensitivity (higher, p < 0.05), and immunosuppressant exposure (higher, p < 0.05), suggesting that clinical phenotype and treatment modulate circulating levels. Another study conducted by the same author demonstrated that lower sRAGE in SLE patients correlated with higher disease activity (SLEDAI, p < 0.01) and organ damage (SDI, p < 0.05) [102]. Another study reported that 94 SLE women with arterial stiffness (Brachial-Ankle Pulse Wave Velocity (baPWV) > 1400 cm/s; n = 35) had significantly lower sRAGE than those with lower stiffness (n = 59, p < 0.05), and multivariable regression confirmed an independent association (β = 1.18, p < 0.01) [107]. Pediatric data further indicate early involvement. Bobek et al. [92] reported sRAGE in juvenile SLE (n = 18), alongside HMGB1 [108]. In contrast, findings are not uniform; Okuyucu et al. [109] examined a mixed rheumatologic cohort (including SLE, RA, and Behçet's disease (BD); n = 60) and found that sRAGE was higher in all patient groups compared with controls (n = 22, p < 0.001), but showed no correlations with C-reactive protein (CRP), erythrocyte sedimentation rate (ESR), or disease activity. Taken together, these findings suggest that decreased sRAGE generally reflects heightened disease burden, yet paradoxical elevations in specific phenotypes highlight its role as a dynamic marker of both inflammation and vascular injury. Current evidence positions sRAGE as a promising adjunct biomarker for SLE monitoring and risk stratification. However, assay heterogeneity, comorbidity effects, and the need for longitudinal validation preclude its use as a stand-alone biomarker at present (Fig. 2). Moreover, total sRAGE assays generally do not distinguish esRAGE from shed cRAGE. Therefore, circulating concentrations reflect the net effects of alternative splicing, receptor shedding, ligand burden, treatment, and clearance rather than a direct quantitative measure of decoy capacity.

**Fig. 2:**
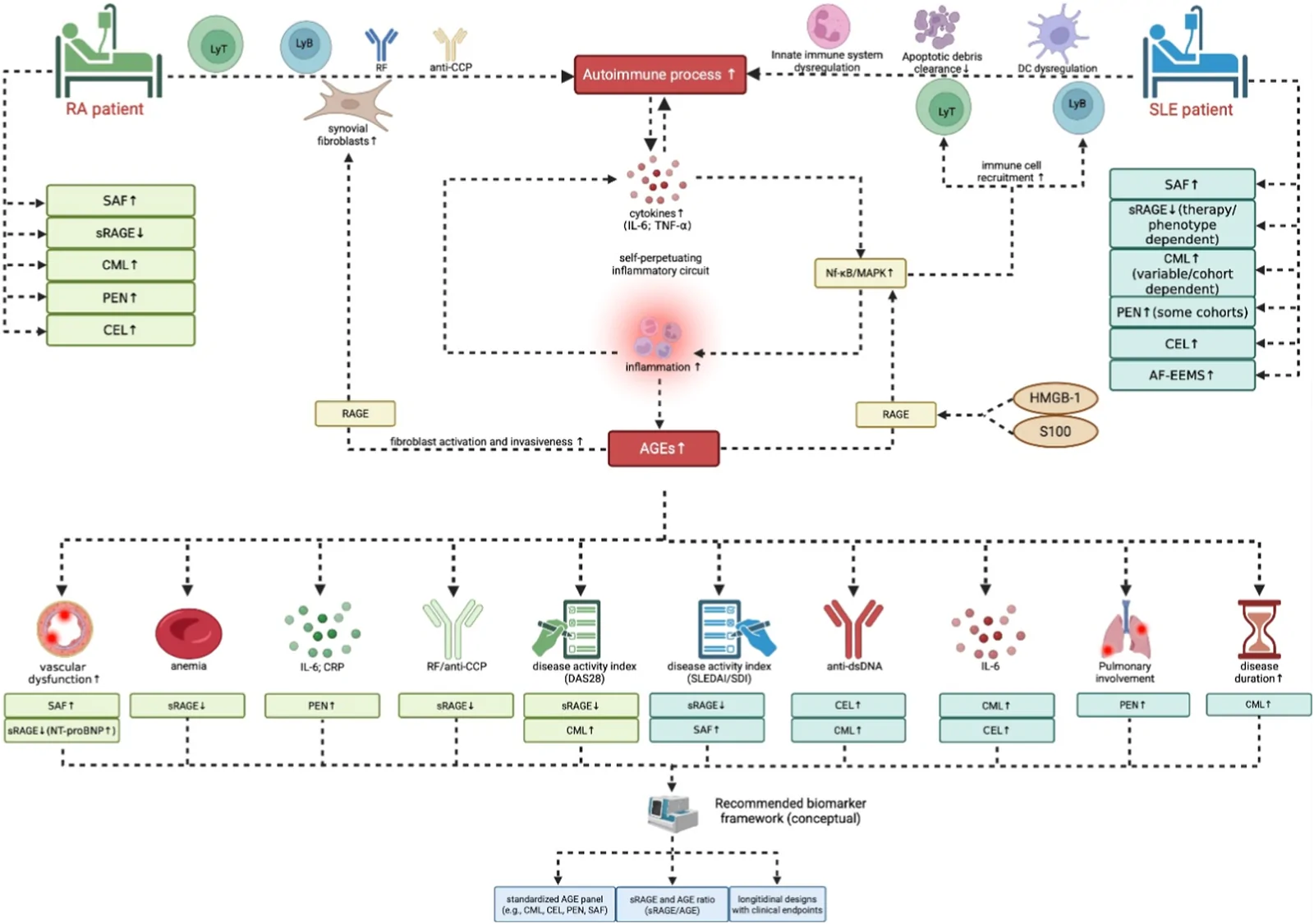
**Biomarker framework of the AGE–RAGE axis in rheumatoid arthritis and systemic lupus erythematosus.** This schematic integrates the proposed role of AGEs and RAGE-related signaling in rheumatoid arthritis (RA) and systemic lupus erythematosus (SLE), together with biomarker patterns discussed in the reviewed literature. In both diseases, AGE accumulation may contribute to a self-perpetuating inflammatory circuit involving cytokine production, immune-cell recruitment, and NF-κB/MAPK activation, whereas disease-associated biomarker signals include changes in SAF, sRAGE, CML, CEL, pentosidine, and AF-EEMS. The section below presents selected clinical correlates and a conceptual framework for evaluating biomarkers based on standardized AGE panels, AGE/sRAGE balance, and long-term clinical follow-up. Abbreviations: AF-EEMS, autofluorescence excitation-emission matrix spectroscopy; AGE, advanced glycation end product; anti-CCP, anti-cyclic citrullinated peptide; CML, carboxymethyllysine; CEL, carboxyethyllysine; CRP, C-reactive protein; DAS28, Disease Activity Score in 28 joints; dsDNA, double-stranded DNA; HMGB1, high mobility group box 1; IL-6, interleukin 6; LyB, B lymphocyte; LyT, T lymphocyte; NF-κB, nuclear factor kappa B; PEN, pentosidine; RA, rheumatoid arthritis; RAGE, receptor for advanced glycation end products; RF, rheumatoid factor; SAF, skin autofluorescence; SDI, Systemic Damage Index; SLE, systemic lupus erythematosus; SLEDAI, Systemic Lupus Erythematosus Disease Activity Index; sRAGE, soluble receptor for advanced glycation end products; TNF-α, tumor necrosis factor alpha.

### 4.2 Rheumatoid arthritis (RA)

RA is a systemic autoimmune disease primarily targeting synovial joints but with well-recognized extraarticular manifestations affecting cardiovascular, pulmonary, hematologic, and neurological systems [110]. The disease course is marked by chronic synovitis, pannus formation, cartilage degradation, and bone erosion, which collectively drive disability and systemic morbidity [110,111]. RA pathogenesis reflects a breakdown of self-tolerance mediated by autoreactive B cells producing rheumatoid factor and anti-citrullinated protein antibodies (ACPA), alongside activated T cells and pathogenic synovial fibroblasts that perpetuate a pro-inflammatory cytokine network dominated by TNF-α, IL-6, and JAK/STAT signaling [56,112,113]. Alarmins such as HMGB1 and S100 proteins amplify inflammation through RAGE and TLR engagement, fueling NF-κB/MAPK activation and establishing a self-perpetuating inflammatory circuit in the synovium [114,115]. In RA, the AGE–RAGE system serves as a bridge between altered metabolic homeostasis and aberrant immunity, illustrating how carbonyl stress and glycation products can amplify synovial damage and promote systemic inflammatory complications [12,53,97].

AGEs accumulate in RA across compartments like synovial fluid, synovium, and skin. Early biochemical studies demonstrated that pentosidine levels are higher in RA than in osteoarthritis (OA) and correlate with systemic inflammatory indices and clinical activity measures, supporting a link between glycation and inflammatory burden [116,117]. Beyond pentosidine, CML has been identified in RA synovial tissue including lining, sublining, endothelium, and on CD68^+^ macrophages and CD45RO^+^ T cells [118]. At the systemic level, serum CML is elevated in RA and associates independently with disease presence and DAS28-defined severity [119]. CEL is also significantly higher in RA serum than in SLE, OA, or healthy controls [120]. Together, these findings suggest that total AGE burden is consistently elevated, whereas subtype specific clinical coupling varies across cohorts and assays. Moreover, ACPA positive individuals at risk of RA exhibit increased tissue AGE accumulation, which can be detectable even before clinical arthritis and may relate to future cardiovascular risk stratification [121]. Mechanistically, carbonyl stress appears to be a proximal driver. As in SLE, MGO is increased in RA and tracks with disease activity, whereas sRAGE may decline as the inflammatory burden increases, consistent with ligand-driven consumption of decoy capacity [122]. Finally, AGE accumulation has functional consequences in the joint niche and beyond. In synovium, RAGE is overexpressed in RA and can be induced in RA fibroblast-like synoviocytes by IL-17/IL-1β [114,123]. This situates AGEs as both structural modifiers (altering matrix resilience or generating potential neoepitopes) and active danger signals that prime innate or adaptive responses. In a Matrigel transwell invasion assay using primary RA fibroblast-like synoviocytes (FLS), stimulation with HMGB1 increased invasiveness to 125% compared to control, while stimulation with AGE-modified albumin increased invasiveness to 150% of control. In both cases, a neutralizing anti-RAGE antibody abrogated the effect, returning invasiveness to near baseline. These findings provide mechanistic evidence that RAGE activation by both inflammatory DAMP ligands and glycation-derived ligands can drive an aggressive, tissue-invasive FLS phenotype, supporting RAGE as a therapeutic target to limit synoviocyte mediated joint destruction [124].

Multiple studies have examined whether AGEs could serve as biomarkers in RA. In one of the earliest reports, Rodríguez-García et al. [125] measured serum pentosidine levels in 50 RA patients and 36 healthy controls, showing significantly higher levels in RA patients (p < 0.001). This finding was reproduced by Hein et al. [117] in a cohort of 82 RA patients, in which serum pentosidine correlated positively with IL-6 (p < 0.001) and CRP (p < 0.001), reinforcing its association with systemic inflammation. However, not all studies confirmed its role as a dynamic marker. In a cohort of 112 RA patients, Senolt et al. [126] observed no significant correlation between pentosidine levels and DAS28 scores (p = 0.48), concluding that pentosidine may better reflect cumulative oxidative stress rather than short-term disease activity. Beyond pentosidine, CML has emerged as a robust candidate marker. In a cross-sectional study of 80 RA patients and 30 healthy controls, Knani et al. [119] reported significantly elevated serum CML levels in RA (p < 0.001). Importantly, CML levels were higher in patients with active RA (DAS28 ≥ 5.1) than in those in remission (p < 0.01). Logistic regression showed that CML level independently predicted RA diagnosis (odds ratio (OR) 1.21; 95% CI 1.05–1.40). Tissue studies further support these observations. Drinda et al. [118] identified CML adducts within RA synovium (n = 15), with staining localized to endothelial cells, macrophages, and infiltrating T cells, confirming in situ AGE deposition at inflammatory sites. Other AGE species, such as CEL, have been less consistent. For example, Vytášek et al. [120] found significantly higher serum CEL in 46 RA patients compared to controls (p < 0.01), but no association with disease duration or activity index, highlighting variability across cohorts. Noninvasive measurements have also been explored. Using SAF, de Groot et al., [127] studied 49 RA patients with controlled disease and 49 age- and sex-matched controls and found higher SAF in RA (p = 0.03). Moreover, SAF correlated with endothelial dysfunction markers (soluble vascular cell adhesion molecule 1 (sVCAM-1), p < 0.01) and reduced arterial elasticity, supporting a role of AGEs in vascular comorbidity. Extending this concept, Hinkema et al. [121] demonstrated increased SAF in 65 ACPA-positive individuals at risk for RA, compared with matched controls (p = 0.02), suggesting that AGE accumulation may precede clinical arthritis and reflect early systemic risk. Finally, AGE modifications may generate neo-epitopes relevant for autoimmunity. Newkirk et al. [128] studied 91 patients with early synovitis and found autoantibodies targeting AGE-modified immunoglobulin G (IgG) in 62% of RA patients, compared with 15% of non-RA synovitis patients (p < 0.001). This suggests that AGE-driven immune alterations could be integrated into early disease processes. The available evidence supports AGE accumulation (mostly pentosidine and CML) as consistently elevated in RA, with measurable associations to inflammatory activity, vascular comorbidity, and autoantibody networks. However, the variability in subtype-specific findings underscores the importance of standardized assays and the need for more prospective studies to define the clinical role of AGEs as reliable biomarkers in RA.

Early evidence from a study on 69 RA patients and 69 matched controls showed significantly lower circulating sRAGE in RA (p < 0.001). Significantly, lower sRAGE correlated with higher CRP (p < 0.01), suggesting deficient anti-inflammatory buffering in active disease [129]. Similarly, Chen et al. [130] examined 101 RA patients and reported that reduced sRAGE was associated with rheumatoid factor, anti-CCP antibodies, and elevated S100 protein levels (all p < 0.05), linking sRAGE deficiency to autoantibody status and vascular risk factors. Prospective studies add prognostic weight. In a longitudinal cohort of 138 women with RA, it was confirmed that lower baseline sRAGE predicted future cardiometabolic events over 10 years (hazard ratio (HR) 1.75; 95% CI 1.21–2.52; p = 0.003) [131]. Similarly, in prospective study of 67 early RA patients, Tam et al. [132] found that reduced sRAGE was independently associated with increased aortic augmentation index (β = −0.32, p = 0.01), linking low sRAGE to early vascular stiffening. Other cohorts explored sRAGE in relation to disease activity, with 82 RA patients demonstrating a negative correlation between sRAGE and DAS28 (r = −0.42, p < 0.001) [133]. In contrast, Okuyucu et al. [109] investigated a mixed rheumatology cohort (60 patients: 25 RA, 20 SLE, 15 Behçet's) and found higher sRAGE across all groups compared to controls (n = 22, p < 0.001), but without correlation to CRP, ESR, or disease activity. Emerging data also explore comorbidities. Heslinga et al. [134] reported in 120 early RA patients that lower sRAGE was linked with higher N-terminal pro-B-type natriuretic peptide (NT-proBNP) levels, suggesting an association with subclinical cardiac stress. Another study investigated 96 RA patients and found decreased sRAGE in those with anemia (p < 0.01), proposing its role as a hematologic marker [135]. At the genetic level, researchers used Mendelian randomization across >400,000 individuals. They demonstrated that higher sRAGE was causally protective against RA risk (OR per 1-standard deviation (SD) increase = 0.82, p = 1.4 × 10^−5^), providing strong evidence for a mechanistic role [136]. Collectively, the data suggest that a lower sRAGE level is generally associated with higher RA activity, autoantibody positivity, vascular dysfunction, and adverse outcomes. Yet, contradictory findings (e.g., elevated sRAGE in mixed cohorts) underscore the dynamic regulation of sRAGE, which is influenced by ligand load, disease stage, and treatment (Fig. 2).

### 4.3 Type 1 diabetes mellitus

Type 1 diabetes mellitus (T1D) is a chronic immune-mediated disorder characterized by progressive dysfunction and destruction of insulin-producing pancreatic β-cells, ultimately resulting in absolute or near-absolute insulin deficiency and persistent hyperglycemia. Its pathogenesis reflects a complex interaction between genetic susceptibility, environmental and immunological factors that culminate in the loss of immune tolerance to β-cell antigens [137]. Autoreactive CD4^+^ and CD8^+^ T cells are central effectors of β-cell injury, while B cells, antigen-presenting cells, innate immune populations and inflammatory mediators contribute to antigen presentation, immune-cell recruitment and propagation of insulitis. Clinically overt disease emerges when residual β-cell function becomes insufficient to maintain glucose homeostasis [138]. Within this pathogenic framework, the AGE–RAGE axis may operate at the intersection of metabolic stress, redox dysregulation and immune activation. This concept requires particular caution because AGE accumulation can be both a consequence of established hyperglycemia and a potential modifier of immune processes preceding overt diabetes. In human T1D, RAGE-expressing T cells display a more activated inflammatory phenotype, and stimulation with the non-AGE RAGE ligand HMGB1 enhances pro-inflammatory T-cell responses [61]. Experimental data further implicate RAGE-dependent signaling in the disruption of immune tolerance in T1D. Extracellular HMGB1 has been shown to compromise regulatory T-cell stability through activation of RAGE- and TLR4-dependent signaling pathways, thereby promoting a pro-inflammatory milieu permissive to autoimmune progression [139]. Complementing these immunological observations, direct exposure of pancreatic β-cells to advanced glycation end products has been demonstrated to induce oxidative and cellular stress, characterized by increased reactive oxygen species generation, mitochondrial dysfunction, impaired insulin biosynthesis and secretion, and activation of pro-apoptotic signaling cascade [140]. Exogenous, diet-derived advanced glycation end products (dAGEs) may represent an additional environmental source of carbonyl and glycoxidative stress relevant to T1D. In non-obese diabetic (NOD) mice, exposure to a diet with a substantially reduced AGE content markedly delayed diabetes development and lowered disease incidence relative to animals receiving an otherwise nutritionally comparable high-AGE diet. The low-AGE environment was associated with attenuated insulitis and a shift toward a less pro-inflammatory T-cell cytokine profile, and protection persisted across subsequent generations maintained under low-glycotoxin conditions [141]. In a complementary study integrating experimental and human genetic data, Forbes et al. found that inhibition of RAGE with alagebrium, one of the AGE cross-link breakers, was associated with reduced diabetes incidence and altered RAGE-related changes during disease progression [142]. Recent work provides particularly compelling evidence for an immunoregulatory role of RAGE axis in T1D. Administration of recombinant sRAGE to NOD mice protected against autoimmune diabetes in independent experimental settings, preserved islet insulin expression and expanded functional regulatory T-cell populations in pancreatic lymph nodes, spleen and pancreatic islets [143].

The biomarker literature in T1D supports a temporal distinction between preclinical islet autoimmunity and established hyperglycemic disease. In prospectively followed children who progressed to T1D, circulating sRAGE was higher than in human leukocyte antigen (HLA)-matched, age-matched, and sex-matched autoantibody-negative controls before seroconversion (P = 0.03), at the appearance of multiple autoantibodies (p = 0.008), and close to diagnosis (p = 0.04), but declined around seroconversion. Importantly, CML did not increase in parallel and was significantly lower in progressors close to diagnosis (p = 0.004), whereas the sRAGE/CML ratio remained higher before seroconversion (p = 0.008) and near diagnosis (p < 0.001)s [144]. In contrast, after T1D is established, evidence for cumulative glycation and glycoxidative stress is considerably stronger. In a cohort of 140 patients with T1D, mean SAF was significantly higher than in 57 non-diabetic controls (2.13 ± 0.55 vs. 1.70 ± 0.27 a. u.) and correlated positively with diabetes duration and glycated hemoglobin (HbA1c) [145]. In analyses of microvascular disease, SAF was associated with retinopathy (OR 2.47, 95% CI 1.26–4.84), nephropathy (OR 3.15, 95% CI 1.34–7.39), neuropathy (OR 3.17, 95% CI 1.46–6.90), and any microvascular complication (OR 2.94, 95% CI 1.46–5.92) in univariate models, although after multivariable adjustment the association remained evident primarily for neuropathy (OR 2.98, 95% CI 0.99–8.90; *P* = 0.05) [146]. Consistently, among 133 patients studied by Chabroux et al., SAF increased with the severity of both retinopathy and nephropathy (both P < 0.001), but after simultaneous adjustment for age, diabetes duration, HbA1c, smoking and other microvascular complications, independent associations persisted for nephropathy and neuropathy but not retinopathy [147]. Direct biochemical analysis further substantiates persistent glycoxidative modification of long-lived proteins: in skin collagen from 96 patients with T1D and 78 controls, fructose-lysine was increased in diabetes (*P* < 0.0001), while age-dependent accumulation of CML and pentosidine remained accelerated after accounting for HbA1c. Methionine sulfoxide accumulated particularly rapidly in patients with vascular complications, and accumulation rates of CML, pentosidine and methionine sulfoxide were all greater in patients with complications than in those without them (all *P* < 0.0001) [148]. Importantly, tissue and circulating AGE measures are not interchangeable. In 68 adults with T1D and 68 matched controls, Llauradó et al. observed higher circulating CML (1.18 vs. 0.96 μg/mL; *P* = 0.008) and SAF (2.10 vs. 1.70 AU; *P* < 0.001), yet only SAF correlated with aortic pulse-wave velocity (*r* = 0.370, *P* = 0.003) and remained independently associated with arterial stiffness after adjustment for conventional cardiovascular risk factors (β = 0.380, *P* < 0.001) [149]. This molecular heterogeneity was further demonstrated in the DIABAGE study of 196 patients with T1D: SAF was associated with retinopathy (OR 1.92, *P* = 0.011), neuropathy (OR 2.02, *P* = 0.04), any microangiopathy (OR 2.83, *P* < 0.0001), coronary disease (OR 3.11, *P* = 0.009), and any macroangiopathy (OR 2.78, *P* = 0.003), whereas circulating pentosidine was preferentially associated with coronary disease (OR 1.61, *P* = 0.01) and macroangiopathy (OR 1.52, *P* = 0.005), and methylglyoxal-derived hydroimidazolone 1 (MG-H1) with nephropathy (OR 1.72, *P* = 0.03). Furthermore, pulse-wave velocity correlated with SAF (*r* = 0.43, *P* < 0.001), pentosidine (*r* = 0.28, *P* < 0.001), and MG-H1 (*r* = 0.16, *P* = 0.031), but not CML (*r* = 0.03, *P* = 0.598) [150]. Prognostic evidence is provided by Nin et al., who followed 339 patients with T1D for a median of 12.3 years: 85 patients (25.1%) experienced a fatal or non-fatal cardiovascular event and 82 (24.2%) died. Each 1-SD increase in a composite plasma AGE score incorporating CML, CEL and pentosidine was associated with a 30% higher risk of incident cardiovascular disease (HR 1.30, 95% CI 1.03–1.66) and a 27% higher risk of all-cause mortality (HR 1.27, 95% CI 1.00–1.62), independently of conventional cardiovascular risk factors, with further adjustment for renal and endothelial dysfunction, low-grade inflammation and arterial stiffness producing little attenuation [151]. Collectively, these data indicate that AGE-related biomarkers in T1D predominantly capture cumulative carbonyl, glycoxidative and tissue injury burden rather than autoimmune activity per se. The absence of progressive circulating CML accumulation before clinical diabetes, contrasted with pronounced tissue AGE accumulation and AGE-specific associations with vascular outcomes after prolonged hyperglycemia, supports a temporally stratified model in which multiligand RAGE-dependent immune signaling may operate during preclinical autoimmunity, whereas subsequent hyperglycemia-driven carbonyl stress increasingly promotes AGE accumulation, extracellular-matrix remodeling and redox-sensitive vascular inflammation.

In 2115 children with newly diagnosed T1D and 316 healthy controls, Salonen et al. found no overall difference in circulating sRAGE (1171 vs. 1153 pg/mL; p = 0.48). However, concentrations were markedly lower in children diagnosed before 2 years of age (1027 vs. 1181 pg/mL in older patients; *P* < 0.001) and in those presenting with diabetic ketoacidosis (1086 vs. 1190 pg/mL; *P* < 0.001), while high-risk HLA-DR3/DR4 and *AGER* rs2070600 genotypes were also associated with lower sRAGE [152]. Longitudinal observations preceding clinical onset further showed that children progressing to T1D had higher sRAGE than autoantibody-negative controls at several time points, but circulating concentrations declined around seroconversion to diabetes-associated autoantibody positivity, whereas CML did not increase concomitantly [144]. Conversely, in children with established T1D, Dettoraki et al. reported higher serum sRAGE than in healthy controls (1430 ± 759 vs. 1158 ± 595 pg/mL; *P* = 0.047), despite markedly lower RAGE protein expression in peripheral blood mononuclear cells (61 ± 46% vs. 102 ± 63%; *P* = 0.0001) and comparable circulating CML concentrations (3.51 ± 1.49 vs. 3.59 ± 1.83 ng/mL). sRAGE also decreased with increasing diabetes duration (*r* = −0.265, *P* = 0.037) [153]. Such apparently discordant findings are mechanistically plausible because insulin treatment itself can modulate the soluble receptor pool: in vitro, insulin increases full-length RAGE and esRAGE expression while simultaneously promoting metalloproteinase-dependent ectodomain shedding. Consistent with this mechanism, Lee et al. demonstrated higher circulating sRAGE, esRAGE and cRAGE in 102 insulin-treated patients with T1D than in 101 controls. cRAGE was increased from a median of 484 (283–796) to 594 (447–812) pg/mL, while serum ADAM10 correlated specifically with cRAGE in T1D (*r* = 0.40, *P* < 0.01) and independently accounted for approximately 21% of its variability, supporting ADAM10-dependent receptor shedding as an important determinant of circulating sRAGE [50]. Renal handling represents an additional major source of variation. Higher sRAGE levels were found in patients with long-standing T1D than in healthy controls (1554 ± 449 vs. 1309 ± 400 pg/mL; *P* = 0.049), with sRAGE independently related to serum creatinine (*r* = 0.32, p < 0.01) [154]. Importantly, prospective outcome studies challenge the assumption that higher circulating sRAGE necessarily denotes greater endogenous protection against RAGE signaling. Among 339 patients with T1D followed for a median of 12.3 years, higher baseline sRAGE independently predicted fatal or non-fatal cardiovascular events (HR 1.90, 95% CI 1.13–3.21) and all-cause mortality (HR 2.12, 95% CI 1.26–3.57). Adjustment for estimated glomerular filtration rate (eGFR) attenuatedthese associations [155]. Similarly, in the FinnDiane cohort of 3100 adults with T1D observed for a median of 9.1 years, 202 deaths occurred, and higher sRAGE remained independently associated with both all-cause and cardiovascular mortality after adjustment for cardiovascular risk factors and chronic kidney disease; eGFR, albuminuria, age, diabetes duration, HbA1c, body mass index (BMI) and insulin dose were themselves independent determinants of circulating sRAGE [156]. The overall inconsistency of individual studies is reflected quantitatively in a recent meta-analysis of nine T1D datasets from eight studies, which identified moderately higher sRAGE in T1D than in non-diabetic controls (standardized mean difference (SMD) 0.45, 95% CI 0.16–0.73; *P* = 0.002), but with substantial heterogeneity between studies (*I*^2^ = 79%). Notably, heterogeneity disappeared in the adult subgroup (*I*^2^ = 0%), in which the pooled effect remained significant (SMD 0.48, 95% CI 0.31–0.65) [157].

Collectively, these data indicate that AGEs and circulating sRAGE integrates multiple processes including genetic regulation, receptor expression and shedding, insulin exposure, renal elimination, inflammatory activity and disease duration, and therefore cannot be interpreted solely as a measure of extracellular ligand-scavenging capacity or as a direct inverse index of tissue AGE–RAGE signaling. From a biomarker perspective, future T1D studies should distinguish total sRAGE from esRAGE and cRAGE and evaluate these isoforms alongside chemically defined AGE species, major non-AGE RAGE ligands, renal function, glycemic exposure and treatment variables to determine whether specific AGEs and sRAGE profiles identify biologically meaningful disease endotypes.

### 4.4 Other autoimmune diseases

In psoriasis, a chronic immune-mediated skin disease driven by interleukin 23/T helper 17 (IL-23/Th17)-centered pathways and reciprocal keratinocyte–immune cell interactions, oxidative stress and systemic low-grade inflammation are well-recognized contributors to disease severity and comorbidity risk. RAGE ligands, including AGEs and S100 proteins, are upregulated in psoriatic skin, suggesting a mechanistic link between metabolic stress and inflammatory signaling [158]. Deletion of the keratinocyte-specific RAGE receptor in mice altered acute skin inflammation and identified epidermal RAGE as a regulator of TNF-α transcription dynamics. This is consistent with its role in maintaining keratinocyte activation and paracrine communication with immune cells in the stroma [159].In a case control study of patients with severe psoriasis and matched healthy controls, both cutaneous and circulating total AGEs, as well as serum pentosidine, were significantly increased in patients, with strong correlations between skin and serum AGE levels and between AGE accumulation and Psoriasis Area and Severity Index (PASI) score [160]. Subsequent targeted biomarker studies confirmed elevations of CML and CEL, along with altered sRAGE concentrations. Although AGE levels decreased during clinical remission, they remained higher than in controls, indicating persistent metabolic stress despite clinical improvement [161]. Consistent with these findings, circulating MGO has been shown to be elevated in psoriasis and to correlate with oxidative stress indices and disease severity [162]. Similar alterations in AGE biology have been reported in multiple sclerosis (MS), a disease characterized by immune-mediated demyelination, oxidative stress, and neurodegeneration, conditions that favor enhanced glycation. In this disease AGE–RAGE signaling contributes to neuroinflammation largely through DAMP that sustain innate activation and facilitate adaptive priming. HMGB1 binds promiscuous receptors including RAGE, Toll-like receptor 2 (TLR2), and TLR4 and promotes cytokine production and T-cell proliferation. In experimental autoimmune encephalomyelitis (EAE), HMGB1 neutralizationameliorated disease, blocked central nervous system (CNS) T-cell infiltration, and reduced systemic CD4^+^ responses to myelin epitopes, with consistent in vitro effects on mouse lymphocytes and stimulated human peripheral blood mononuclear cells (PBMCs) [163]. In addition, deficiency altered T-cell activation and cytokine outputs, supporting a plausible route by which RAGE ligation may modulate encephalitogenic T-cell phenotypes [20,164]. Elevated circulating AGEs and AGE-modified proteins have been detected in MS patients compared with healthy controls, and AGE deposition has been observed within MS brain tissue, particularly in active lesions [165]. High-resolution LC–MS/MS profiling demonstrated increased plasma CEL concentrations in disease-modifying therapy, naïve MS patients, with levels decreasing substantially following treatment and correlating with relapse frequency [166]. enzyme-linked immunosorbent assay (ELISA)-based studies have similarly reported higher circulating CML levels in MS [167]. Furthermore, multiple studies indicate that MS is associated with altered expression of the receptor RAGE and glyoxalase 1 (Glo1), the main enzyme responsible for MGO detoxification [168]. In inflammatory bowel disease (IBD), RAGE ligands (AGEs, HMGB1, S100/calgranulins) are enriched in inflamed mucosa and can amplify epithelial and myeloid inflammatory programs through NF-κB/MAPK/JAK–STAT signaling [169]. In vivo, pharmacologic RAGE inhibition with FPS-ZM1 (RAGE-specific antagonist which binds to the V domain of receptor), improved gut damage during experimental enteritis and colitis, indicating functional pathway relevance and tractability [169]. Ligand-focused mouse work further supports a RAGE-dependent mechanism at the epithelial interface: soluble S100A4 increased bacterial adherence to intestinal epithelial cells and aggravated colitis development, and these effects were abolished by FPS-ZM1 [170]. Moreover, evidence supports AGE accumulation and RAGE-pathway activation in IBD. Urinary pentosidine correlated with ulcerative colitis activity [171]. In a larger study (Crohn's disease n = 56; ulcerative colitis n = 60; healthy controls n = 113), sRAGE levels were altered across different IBD, with higher sRAGE in ulcerative colitis, while biologic therapy in Crohn's disease was associated with lower sRAGE [172]. Emerging evidence indicates that AGE/RAGE signaling may have predictive value for postoperative recurrence in Crohn's disease [173,174]. Notably, RAGE expression is significantly upregulated in inflamed intestinal tissues of Crohn's disease patients [175].

Taken together, the available evidence indicates that increased AGE accumulation, enhanced availability of RAGE ligands, and altered sRAGE buffering capacity are recurring features across autoimmune diseases. Although the specific clinical manifestations vary by organ system, the convergence of oxidative stress, carbonyl stress, and RAGE signaling suggests a shared inflammatory amplification loop that may link metabolic stress to chronic immune activation.

## 5 Anti-glycation interventions in autoimmune diseases

Compounds that inhibit AGE formation or RAGE signaling represent promising therapeutic strategies in autoimmune diseases. These agents exert their effects through multiple mechanistic pathways, including inhibition of early-stage glycation by suppressing AGE precursors, carbonyl scavenging or trapping, chelation of metal ions, disruption of AGE crosslinks, or direct RAGE blockade [176,177]. At present, the majority of evidence supporting the efficacy of AGE inhibitors derives from in vitro experiments, with only a limited subset of compounds having advanced to clinical evaluation (Fig. 3). To provide a more comprehensive overview, this section discusses both agents already evaluated in autoimmune settings and established antiglycation compounds for which evidence currently derives mainly from diabetes, metabolic disease, or experimental models.

**Fig. 3:**
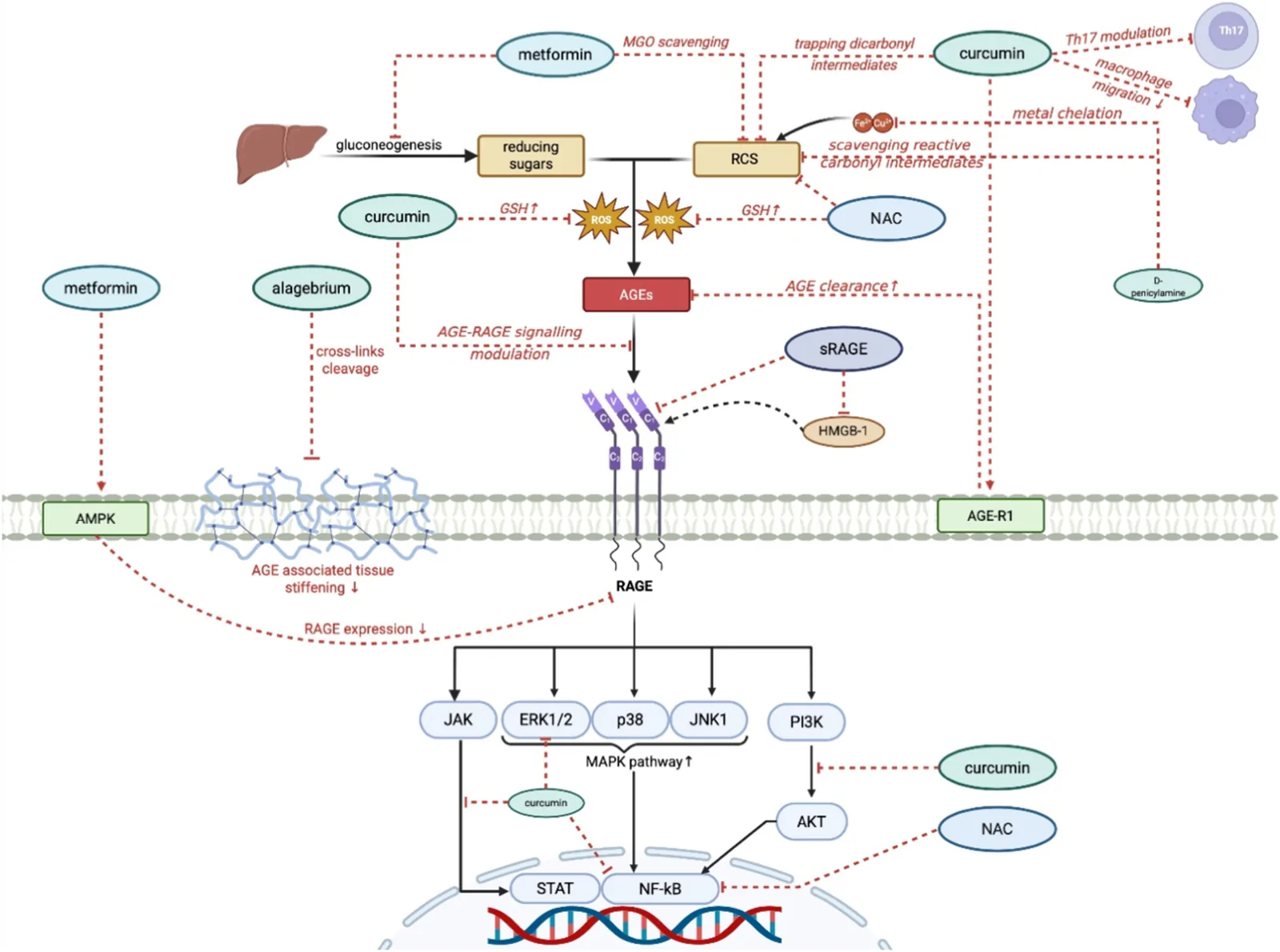
**Conceptual overview of anti-glycation strategies targeting the AGE–RAGE axis in autoimmune diseases.** This figure summarizes the main mechanisms by which selected anti-glycation or RAGE-modulating interventions may interfere with AGE-driven inflammation. Proposed actions include reduction of glycation substrate availability, scavenging of methylglyoxal and other reactive carbonyl intermediates, attenuation of oxidative stress, modulation of RAGE expression and downstream signaling, enhancement of AGE clearance, and cleavage of AGE-related cross-links. The scheme includes metformin, curcumin, N-acetylcysteine (NAC), d-penicillamine, alagebrium, and soluble RAGE (sRAGE) as representative examples discussed in this review. Abbreviations: AGE, advanced glycation end product; AGE-R1, advanced glycation end product receptor 1; AKT, protein kinase B; AMPK, AMP-activated protein kinase; C_1_, C_1_ domain of RAGE; C_2_, C_2_ domain of RAGE; ERK1/2, extracellular signal-regulated kinase 1/2; GSH, glutathione; HMGB1, high mobility group box 1; JAK, Janus kinase; JNK1, c-Jun N-terminal kinase 1; MAPK, mitogen-activated protein kinase; MGO, methylglyoxal; NAC, N-acetylcysteine; NF-κB, nuclear factor kappa B; PI3K, phosphoinositide 3-kinase; RAGE, receptor for advanced glycation end products; RCS, reactive carbonyl species; ROS, reactive oxygen species; sRAGE, soluble receptor for advanced glycation end products; STAT, signal transducer and activator of transcription; V, V domain of RAGE.

Curcumin, a polyphenolic compound isolated from *Curcuma longa*, has been extensively studied for its anti-glycation and anti-oxidative pharmacology [178]. Mechanistically, curcumin directly inhibits the non-enzymatic formation of AGEs by trapping reactive dicarbonyl intermediates, thereby reducing carbonyl stress and subsequent AGE generation. This mechanism is accompanied by reductions in oxidative damage and pro-inflammatory cytokine expression [179]. Curcumin also modulates AGE–RAGE signaling at the receptor and downstream pathways. In hepatic stellate cells exposed to AGEs, curcumin interrupts AGE-induced signaling cascades, including Janus kinase 2/signal transducer and activator of transcription 3 (JAK2/STAT3) and PI3K/AKT, that drive upregulation of RAGE and suppression of AGE-R1, effectively normalizing receptor expression and attenuating oxidative stress via enhanced nuclear factor erythroid 2-related factor 2 (Nrf2) activity and glutathione elevation [178]. Moreover, curcumin can increase expression of AGE-clearance receptors (e.g., AGE-R1) while inhibiting pro-fibrogenic signaling and ERK activity [180]. In vivo modulation of the inflammatory pathway is supported by studies showing that oral nanocurcumin suppressed NFκB signaling and macrophage migration in an LPS-driven inflammatory model [181]. In clinical evidence, a nanocurcumin study in ankylosing spondylitis demonstrated its ability to modulate Th17 cell function, which is relevant to autoimmune pathology. Although the record does not fully detail the numerical immunophenotyping results, the study's premise highlights a targeted inflammatory T-cell axis intervention, supporting the potential immunomodulatory effects of curcumin in autoimmune contexts [182]. Additionally, a systematic review of curcumin in rheumatic diseases reports small interventional studies in rheumatoid arthritis and lupus nephritis [183]. A separate review summarizes curcumin's immunomodulatory effects across systemic autoimmune diseases and notes clinically relevant outcomes in juvenile idiopathic arthritis when combined with standard therapies [184]. However, these studies do not measure AGE adducts as pharmacodynamic endpoints, limiting the attribution of clinical outcomes to anti-glycation mechanisms versus broader anti-inflammatory effects. A translational pathway for anti-glycation interventions in autoimmunity is drug repurposing, which involves identifying existing agents in autoimmune care that exhibit anti-glycation activity.

d-penicillamine is a low-molecular-weight thiol with multimodal anti-glycoxidative activity. Mechanistically, it can scavenge reactive carbonyl intermediates (including glycation-derived dicarbonyls such as methylglyoxal) and chelate transition metals, thereby limiting metal-catalyzed oxidation steps that accelerate glycoxidation [185,186]. In cellular and protein glycation models, d-penicillamine attenuated high-glucose driven glycoxidative injury and reduced AGE-related damage, consistent with carbonyl-trapping and chelation-mediated suppression of glycoxidation rather than direct antagonism of RAGE signaling [185,187]. Comparative in vitro screening assays also report antiglycation activity for d-penicillamine (e.g., bovine serum albumin (BSA)–MGO systems), although effect size can be assay- and readout-dependent, reflecting differences in glycation chemistry, oxidation conditions, and endpoint quantification [188]. d-penicillamine is clinically established for rheumatoid arthritis, providing a practical interface for its potential use in anti-glycation roles related to autoimmune treatment. However, no animal models of autoimmunity or clinical trials explicitly examining d-penicillamine for AGE lowering or modulation of the AGE–RAGE pathway are currently documented.

Alagebrium (ALT-711) is a small-molecule agent developed to selectively cleave pre-existing AGE-derived protein cross-links within long-lived extracellular matrix proteins, thereby aiming to reverse AGE-associated tissue stiffening. Early clinical investigations, including studies in arterial stiffness and diastolic heart failure, demonstrated improvements in selected hemodynamic and functional parameters, supporting the biological plausibility of AGE cross-link reversal as a therapeutic strategy [189,190]. Conceptually, crosslink disruption could be relevant to autoimmune phenotypes characterized by vascular dysfunction and tissue remodeling. However, the absence of autoimmune animal models or interventional trials means this remains a hypothesis. Testing this hypothesis would require rigorous designs coupling valid endpoints with AGE panels and RAGE-axis biomarkers.

Metformin (1,1-dimethylbiguanide) is a first-line antihyperglycemic agent whose relevance to AGEs biology derives from combined upstream metabolic effects and downstream molecular actions. At the metabolic level, metformin reduces hepatic gluconeogenesis and improves peripheral insulin sensitivity, thereby lowering chronic hyperglycemia and reducing substrate availability for non-enzymatic protein glycation [191]. Beyond these indirect effects, metformin has been shown to directly scavenge the MGO, forming stable metformin–MGO adducts [192,193]. At the signaling level, metformin attenuates AGE-induced inflammatory responses by activating AMP-activated protein kinase (AMPK), which, in turn, suppresses RAGE expression and downstream NF-κB signaling, leading to reduced production of pro-inflammatory cytokines in macrophages, endothelial cells, and renal tubular cells exposed to AGEs [194]. This convergence further supports a mechanistic linkage between these agents and attenuation of AGE-driven oxidative stress and inflammatory signaling [195,196]. Metformin is supported by mechanistic links to AGE biology and extensive preclinical evidence of immunometabolic modulation [194,197]. In autoimmune models, metformin attenuated CNS autoimmunity in experimental autoimmune encephalomyelitis [198], ameliorated experimental autoimmune arthritis, with reportedeffects on Th17/Treg balance and osteoclastogenesis [199], and reduced immune infiltration in Sjögren's syndrome models [200]. This compound is generally positioned as an immunomodulatory candidate across autoimmune indications [201]. From an anti-glycation perspective, it is necessary to conduct autoimmune trials with validated glycation and RAGE-axis pharmacodynamic endpoints and demonstrate mechanistic engagement alongside clinical outcomes.

N-acetylcysteine (NAC) is a thiol-containing compound with the ability to attenuate carbonyl stress, restore intracellular redox balance, and suppress AGE-induced inflammatory signaling [202]. NAC serves as a cysteine donor for intracellular glutathione (GSH) synthesis, thereby enhancing endogenous antioxidant capacity and limiting glycoxidative amplification of AGE formation [203]. At the signaling level, NAC has been shown to suppress AGE–RAGE–mediated activation of NF-κB, leading to reduced expression of pro-inflammatory cytokines, adhesion molecules, and oxidative enzymes in endothelial cells and other AGE-responsive cell types [[204], [205], [206]]. Experimental evidence further demonstrates that chronic NAC supplementation in animal models diminishes plasma AGE levels, reduces lipid peroxidation, and mitigates macrophage infiltration and inflammation in adipose tissue exposed to AGE-albumin, consistent with a role in blunting AGE-induced oxidative damage and its metabolic consequences [207]. Complementary in vitro studies reveal that NAC suppresses AGE-induced apoptosis and ROS generation in human stem cell [208]. Nevertheless, NAC's broad antioxidant and immunomodulatory actions are major confounders. Therefore, the clinical efficacy of anti-glycation therapies remains speculative without controlled trials incorporating standardized AGE adduct panels and RAGE-axis biomarkers.

sRAGE works as a circulating decoy receptor, sequestering a broad spectrum of RAGE ligands, AGEs, S100/calgranulin proteins, HMGB1, amyloid-β, and other damage-associated molecular patterns, thereby preventing their interaction with cell-surface RAGE and disrupting the inflammatory loop that sustains chronic inflammation [209]. Endogenous sRAGE exists in at least two forms: cleaved sRAGE, generated by proteolytic shedding of membrane RAGE by metalloproteinases (e.g., ADAM10, MMPs), and endogenous secretory RAGE (esRAGE), produced via alternative splicing of RAGE gene. Experimental administration of recombinant sRAGE in animal models has consistently demonstrated attenuation of AGE- and ligand-driven inflammation, including reductions in oxidative stress, NF-κB activation, cytokine expression, vascular dysfunction, and tissue injury in models of diabetes, atherosclerosis, nephropathy, and neuroinflammation [210,211]. In autoimmune cohorts, sRAGE has been discussed as a marker and potential modifier of HMGB1–RAGE–NF-κB signaling, including in antiphospholipid syndrome [212]. As with other discussed interventions, there are currently no in vivo studies or clinical trials that directly link sRAGE to validated anti-glycation outcomes. Thus, the sRAGE-based therapeutic approach remains speculative.

Several additional antiglycation strategies have been investigated predominantly in diabetes, obesity or vascular aging, but have not been adequately evaluated in autoimmune diseases. Aminoguanidine (pimagedine) acts as a nucleophilic carbonyl trap. In the ACTION I randomized, double-masked trial involving 690 patients with T1D, nephropathy, and retinopathy, pimagedine did not significantly reduce the primary endpoint of doubling of serum creatinine (26% with placebo vs. 20% with aminoguanidine; p = 0.099). Although decline in estimated glomerular filtration rate, proteinuria, and retinopathy progression showed favorable secondary signals, high-dose treatment was associated with cases of glomerulonephritis [213]. Pyridoxamine inhibits glycation through carbonyl scavenging, metal-chelating, and antioxidant actions. In combined 24-week phase 2 studies including patients with type 1 and type 2 diabetes and overt nephropathy, pyridoxamine reduced the rise in serum creatinine in the merged analysis and was accompanied by decreases in CML and CEL, although albuminuria was unchanged [214]. These trials demonstrate human pharmacological tractability of AGE formation, but they targeted diabetic complications rather than autoimmune mechanisms, and therefore cannot be taken as evidence of immune disease modification. Benfotiamine, a lipid-soluble thiamine derivative, represents an indirect approach to suppression of dicarbonyl stress by increasing transketolase activity and thereby diverting excess glycolytic intermediates away from pathways generating methylglyoxal and downstream AGEs. In 13 patients with type 2 diabetes, short-term benfotiamine treatment (1050 mg/day for 3 days) prevented the marked impairment of flow-mediated dilation and reactive hyperemia induced by an AGE-rich meal and attenuated the associated increases in methylglyoxal, AGE-related and oxidative-stress markers [215]. However, this acute metabolic effect did not translate into demonstrable sustained AGE lowering in a larger randomized trial with 12 weeks of benfotiamine (900 mg/day) in patients with type 2 diabetes and early nephropathy produced no significant changes in plasma or urinary CML, CEL or MG-H1, or in biomarkers of endothelial dysfunction and inflammation [216]. Carnosine (β-alanyl-l-histidine) provides a complementary strategy through reactive-carbonyl sequestration, metal chelation and antioxidant activity, and extensive experimental evidence indicates inhibition of both glycation and glycoxidation [217]. In a double-blind randomized trial involving 54 patients with type 2 diabetes, 1 g/day carnosine for 12 weeks reduced circulating CML by 91.8 ng/mL relative to placebo and decreased pentosidine by 2.8 ng/mL from baseline, accompanied by reductions in fasting glucose and HbA1c, whereas sRAGE remained unchanged [218]. These findings provide direct human evidence of modulation of AGE burden, although the small sample size and pleiotropic metabolic actions of carnosine preclude attribution of the clinical effects specifically to antiglycation activity. Targeting the glyoxalase system itself represents a particularly attractive mechanism-based strategy. An optimized trans-resveratrol–hesperetin combination developed as a Glo1 inducer increased Glo1 expression and activity by 27% in highly overweight subjects, while reducing plasma methylglyoxal by 37% and methylglyoxal-derived protein glycation by 14%. These biochemical effects were accompanied by improved insulin sensitivity, glycemic control and vascular function [219]. Unlike nonspecific antioxidant supplementation, this approach therefore provides evidence of pharmacodynamic engagement of a defined dicarbonyl-detoxification pathway, although its clinical evaluation has so far been restricted largely to metabolically compromised populations. Alpha-lipoic acid (ALA) represents a redox-active approach with potential secondary effects on glycation [220]. Its principal pharmacology is antioxidant and thiol-redox modulation rather than selective carbonyl trapping. In a recent six-month clinical study in patients with type 2 diabetes and neuropathy, ALA treatment was associated with a modest but statistically significant reduction in circulating AGE levels, whereas sRAGE was unchanged [221]. This observation provides human evidence that ALA can influence AGE burden, but it derives from metabolic disease rather than autoimmunity and does not establish AGE/RAGE target engagement as the mechanism underlying its clinical effects. Natural polyphenols provide another broad class of candidate antiglycation agents. Quercetin can directly trap methylglyoxal and glyoxal and inhibit AGE formation in albumin–dicarbonyl systems, providing a defined biochemical mechanism [222]. Resveratrol has been reported to suppress AGE formation and attenuate AGE–RAGE-associated downstream signaling across a heterogeneous, predominantly preclinical literature. A systematic review of 29 studies identified effects involving RAGE, NF-κB, oxidative stress, inflammatory signaling, peroxisome proliferator-activated receptor gamma (PPARγ) and transforming growth factor beta (TGF-β) pathways [223]. Both compounds are pleiotropic antioxidants and signaling modulators, and robust autoimmune clinical trials incorporating direct AGE/RAGE pharmacodynamic endpoints are lacking. They should therefore be regarded as mechanistically promising rather than established antiglycation therapies for autoimmune disease. Collectively, these compounds demonstrate that glycation can potentially be targeted at multiple levels, including glycolytic precursor flux, reactive-dicarbonyl scavenging, endogenous glyoxalase-mediated detoxification and modification of established extracellular-matrix cross-links. Nevertheless, their evidence base derives predominantly from metabolic disease and experimental systems, and none has yet demonstrated that pharmacological attenuation of carbonyl or AGE burden modifies autoimmune disease activity. They should therefore be considered mechanistically informative translational candidates rather than established antiglycation therapies for autoimmunity.

## 6 Conclusions and future perspectives

The AGE–RAGE axis represents a mechanistically coherent, but biologically heterogeneous, interface between glycoxidative stress and immune dysregulation. Across the autoimmune conditions discussed, increased AGE burden and altered RAGE profiles are associated with inflammatory activity, tissue and vascular injury, and clinical phenotype. However, interpretation is strongly influenced by the AGE species measured, analytical matrix, disease stage, renal function, treatment exposure, and the contribution of non-AGE RAGE ligands (Table 2). T1D particularly illustrates this temporal complexity: multiligand RAGE-dependent immune signaling may operate during preclinical autoimmunity, whereas sustained hyperglycemia subsequently accelerates dicarbonyl stress, AGE accumulation, and glycoxidative tissue injury. Multiple therapeutic entry points exist, including inhibition of AGE formation, reactive-carbonyl scavenging, enhancement of endogenous dicarbonyl detoxification, redox modulation, RAGE interference, and disruption of established AGE-related cross-links. Expanded evidence from aminoguanidine, pyridoxamine, alpha-lipoic acid, benfotiamine, carnosine, polyphenols, and glyoxalase-inducing strategies demonstrates that carbonyl and AGE pathways are pharmacologically tractable, but most evidence derives from diabetes, metabolic disease, or experimental models rather than autoimmune clinical trials. Consequently, even when an intervention improves clinical outcomes, attribution to antiglycation biology remains inferential unless direct AGE/RAGE pharmacodynamic target engagement is demonstrated. Establishing the therapeutic relevance of the AGE–RAGE axis in autoimmunity will therefore require longitudinal, mechanistically grounded studies using chemically defined AGE measurements, isoform-resolved sRAGE profiling, and clinically meaningful immune and vascular outcomes.

**Table 2:** Disease-specific biomarker signals for AGEs and sRAGE across autoimmune diseases.

Disease/setting	AGE-related findings (directionality and measurement)	sRAGE findings	Clinical importance	Key references
Systemic lupus erythematosus (SLE)	↑ AGE-related fluorescence (AF-EEMS) and/or ↑ total serum AGEs; skin autofluorescence (SAF) elevated; subtype results (CML/CEL/pentosidine) vary across cohorts	sRAGE decreased; therapy and phenotype may modulate levels	SAF and/or AGEs may correlate with disease activity (SLEDAI) and (SDI); heterogeneity emphasizes assay standardization	[94,98,99,101,102]
Rheumatoid arthritis (RA)	↑ pentosidine (serum/synovial compartments); ↑ CML in synovial tissue and serum; ↑ CEL reported; SAF elevated in some cohorts including “at-risk” ACPA + individuals	sRAGE decreased; associations reported with inflammation, autoantibodies, and vascular parameters	Mixed evidence for pentosidine as a dynamic activity marker; CML more consistently associated with RA presence/severity; low sRAGE may correlates with cardiometabolic events	[116,118,119,121,125,129,131]
Type 1 diabetes (T1D)	Preclinical CML does not progressively increase; established T1D shows ↑ SAF and accumulation of tissue and circulating AGE species, including CML, pentosidine and MG-H1	Highly variable and stage-dependent; altered around seroconversion and disease onset; influenced by *AGER* genotype, insulin treatment, receptor shedding and renal function	AGE markers primarily reflect cumulative glycemic/glycoxidative injury and vascular complication burden rather than autoimmune activity; sRAGE has limited stand-alone biomarker specificity	[50,[144], [145], [146], [147], [148], [149], [150], [151], [152], [153], [154], [155], [156], [157],^224^]
Psoriasis	↑ total AGEs and pentosidine; ↑ CML/CEL; levels may remain higher than controls even in remission	sRAGE altered (reported increases in some datasets)	Correlates with disease severity (e.g., PASI) and systemic oxidative stress	[[160], [161], [162]]
Multiple sclerosis (MS)	↑ circulating AGE adducts (e.g., CML/CEL); AGE deposition in lesions; therapy may reduce some AGE signals	sRAGE decreased	Potential links to relapse frequency and treatment response	[165,166,225]
Inflammatory bowel disease (IBD)	Urinary pentosidine tracks disease activity in some cohorts	sRAGE altered; reported higher in ulcerative colitis (UC) than Crohn's disease (CD) and correlating with activity	Suggests disease- and phenotype-specific regulation	[171,172]

ACPA – Anti-Citrulinated Protein Antibody; AF-EEMS – Autofluorescence-Excitation Emission Matrix Spectroscopy; AGE – Advanced Glycation End Product; CEL – Carboxyethyllysine; CML – Carboxymethyllysine; PASI – Psoriasis Area and Severity Index; SAF – Skin Autofluorescence; SDI – Systemic Damage Index; SLEDAI - Systemic lupus erythematosus Disease Activity Index; sRAGE – Soluble Receptor for Advanced Glycation End-products.

Future clinical translation of anti-glycation strategies in autoimmunity will require a more standardized framework. Trials should prespecify harmonized biomarker panels that include a minimal set of validated AGE adducts. To improve signal detection and biological interpretability, enrollment and stratification should be guided by baseline AGE burden, RAGE-ligand load, and vascular involvement, thereby enriching for patients in whom the AGE–RAGE axis is most likely to be a causal driver. Trial architecture should explicitly support causal inference through prespecified mediation analyses linking pharmacodynamic biomarker shifts to clinical outcomes and through comparator strategies capable of separating true anti-glycation engagement from pleiotropic anti-inflammatory effects. In parallel, rational combination approaches targeting multiple nodes of the cascade (e.g., carbonyl trapping paired with RAGE antagonism) warrant evaluation, provided safety and immunological trade-offs are prospectively monitored. Finally, for decoy receptor modalities, early translational development should explicitly address feasibility constraints, pharmacokinetics, pharmacodynamics, and immunogenicity risk.

## CRediT authorship contribution statement

**Szymon Drygała:** Conceptualization, Data curation, Writing – original draft, Writing – review & editing. **Roman Cemaga:** Writing – review & editing. **Mateusz Maciejczyk:** Conceptualization, Data curation, Supervision, Writing – original draft, Writing – review & editing.

## Declaration of generative AI and AI-assisted technologies in the manuscript preparation process

During preparation of this work, the authors used ChatGPT for language editing and proofreading and for refinement of graphical-abstract text. The authors reviewed and edited all outputs and take full responsibility for the content of the manuscript.

## Funding

This research did not receive any specific grant from funding agencies in the public, commercial, or not-for-profit sectors.

## Declaration of competing interest

The authors declare that they have no known competing financial interests or personal relationships that could have appeared to influence the work reported in this paper.

## Data Availability

No data was used for the research described in the article.
